# Shifting goals: effects of active and observational experience on infants’ understanding of higher order goals

**DOI:** 10.3389/fpsyg.2015.00310

**Published:** 2015-03-23

**Authors:** Sarah A. Gerson, Neha Mahajan, Jessica A. Sommerville, Lauren Matz, Amanda L. Woodward

**Affiliations:** ^1^University of St Andrews, Saint AndrewsUK; ^2^University of Maryland, College Park, MDUSA; ^3^Donders Institute for Brain, Cognition and Behaviour, Radboud University Nijmegen, NijmegenNetherlands; ^4^Portland State University, Portland, ORUSA; ^5^University of Washington, Seattle, WAUSA; ^6^University of Chicago, Chicago, ILUSA

**Keywords:** action understanding, action perception links, means-end actions, social cognition, motor learning, infant cognition

## Abstract

Action perception links have been argued to support the emergence of action understanding, but their role in infants’ perception of distal goals has not been fully investigated. The current experiments address this issue. During the development of means-end actions, infants shift their focus from the means of the action to the distal goal. In Experiment One, we evaluated whether this same shift in attention (from the means to the distal goal) when learning to produce multi-step actions is reflected in infants’ perception of others’ means-end actions. Eight-months-old infants underwent active training in means-end action production and their subsequent analysis of an observed means-end action was assessed in a visual habituation paradigm. Infants’ degree of success in the training paradigm was related to their subsequent interpretation of the observed action as directed at the means versus the distal goal. In Experiment Two, observational and control manipulations provided evidence that these effects depended on the infants’ active engagement in the means-end actions. These results suggest that the processes that give rise to means-end structure in infants’ motor behavior also support the emergence of means-end structure in their analysis of others’ goals.

## Introduction

Human infants are highly attentive and responsive to their social partners. They are also cognitively engaged with them. Research over the last decade has revealed that infants encode others’ behavior not just as physical motions through space but rather as actions structured by goals (see Meltzoff, 2007; Woodward et al., 2009 for reviews). This sensitivity to the goal structure of action is a cornerstone of social cognition, providing the foundation for social learning (Tomasello, 1999; Baldwin and Moses, 2001) and theory of mind (Wellman et al., 2004, 2008) in early childhood. Given the importance of infants’ goal sensitivity, recent research has investigated the factors that support its development during infancy. One insight from this research is the finding that infants’ own experience acting in goal-directed ways seems to inform their sensitivity to others’ action goals (e.g., Sommerville et al., 2005, 2008). In the studies reported here, we investigate this process, asking whether and how infants’ own actions may inform their sensitivity to distal goals in others’ actions.

At a basic level of analysis, adults understand actions as directed at the objects that are the proximal targets of the action. For example, imagine a man reaching across a crowded countertop to grasp a spoon. Adults view this action as organized by the relation between the man and the spoon, rather than in terms of its other perceivable attributes (e.g., the reach trajectory, speed of reach, etc.). Infants perceive this action in the same manner by the time they are 6 months of age. For example, when infants in a visual habituation experiment view a repeated goal-directed action (e.g., a person grasping a toy) they subsequently show selective recovery (longer looking) to test events in which the relation between the person and her goal is disrupted compared to trials on which the person’s movements differ but her goal remains the same (e.g., Woodward, 1998; Biro and Leslie, 2006; Brandone and Wellman, 2010; Thoermer et al., 2013). Infants’ selective attention to the goal structure of others’ actions has also been revealed using measures of behavioral imitation, visual anticipation, and neural activity (e.g., Hamlin et al., 2008; Southgate et al., 2009; Cannon and Woodward, 2012; Krogh-Jespersen and Woodward, 2014).

**FIGURE 1:**
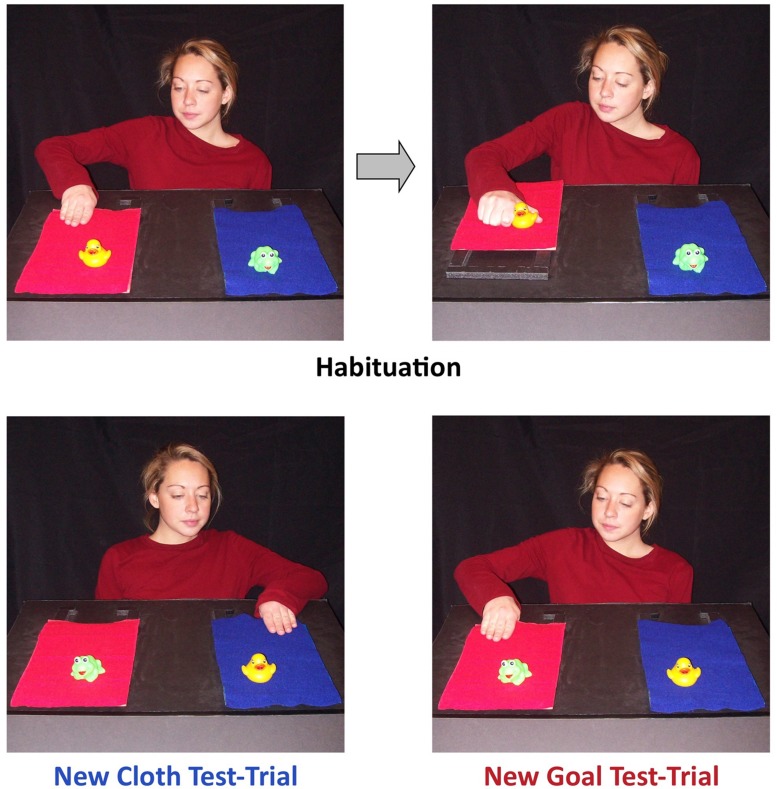
**Habituation paradigm used in Sommerville and Woodward (2005) and current experiments**.

Perceiving meaningful structure in others’ actions requires more than the ability to encode single actions as goal-directed. Individual actions are often assembled in service of distal goals, and when this occurs, a simple action, like grasping a spoon, can be viewed as directed at a distal goal, such as stirring a pot of soup or feeding a baby. To analyze these downstream goals, the perceiver must shift focus from the proximal relations between agents and the objects they touch, to the distal relations between agents and their downstream goals. Recent findings have shown that infants engage in this kind of action analysis by 12 months of age. In one experiment, Sommerville and Woodward (2005) habituated 12-months-old infants to events like the ones depicted in the top panels of **Figure 1**. A woman grasped a cloth and pulled it toward her, thereby drawing near a toy that sat at its far edge. She then grasped the toy. The question of interest was whether infants viewed the woman’s actions on the cloth as directed at the cloth or at the toy. To address this question, Sommerville and Woodward (2005) showed infants test events in which the toys’ locations were reversed (see the lower panels of **Figure 1**) and the woman either reached for the cloth on which she had previously acted which now held a new toy (*new-toy trials*), or the other cloth, which now held the toy she had previously attained (*new-cloth trials*). Twelve-months-old infants looked longer at new-toy trials than new-cloth trials, indicating that they interpreted the woman’s actions on the cloth as directed at the toy; younger infants, 10-months-olds, did not respond systematically in this procedure (see Woodward and Sommerville, 2000; Biro et al., 2011 for related findings).

Critically, 12-months-old infants in this experiment (Sommerville and Woodward, 2005) used the causal structure of the event to interpret its means-end structure. A control group of infants was shown events that mimicked the surface structure of the events depicted in **Figure 1**, but which differed in causal structure because the toy sat next to the cloth rather than on it. In this condition, infants saw the experimenter grasp the cloth, pull the cloth and then grasp the toy, just as in the experimental condition. The act of pulling the cloth reliably preceded and was associated with grasping the toy, but nevertheless, infants in this condition did not interpret the cloth-grasp as directed at the toy (see Woodward and Sommerville, 2000 and Henderson and Woodward, 2011 for similar findings). That is, infants analyzed the same action, grasping the cloth, differently depending on whether it was causally related to attaining a distal goal. Thus, by 12 months, but possibly not before this time, infants are able to look beyond the proximal connections between agents and objects to discern distal goals.

Recent findings indicate that infants’ sensitivity to the goal structure in others’ actions is correlated with and affected by their own motor experience. These effects have principally been documented in studies of infants’ production and perception of simple goal-directed actions, like reaching for a toy (Sommerville et al., 2005; Kanakogi and Itakura, 2010; Libertus and Needham, 2010; Daum et al., 2011; Loucks and Sommerville, 2012; Gerson and Woodward, 2014a,b). For example, Sommerville et al. (2005) found that 3-months-old infants who were trained to use Velcro-covered mittens to apprehend toys subsequently responded systematically to the goal structure of another person’s reaching actions, but infants who did not undergo training did not (see also Libertus and Needham, 2010; Rakison and Krogh, 2011; Gerson and Woodward, 2014a). These behavioral findings are also consistent with recent neural evidence of shared representations between action production and perception in the brain (Rizzolatti and Craighero, 2004; Gerson et al., 2014).

In the case of simple actions, like grasping, motor experience may yield relatively concrete evidence about the way in which a particular action is organized with respect to goals. But understanding downstream goals requires a more flexible analysis of particular actions as potentially directed at distal goals rather than their proximal targets. Research regarding the role of experience in the understanding of means-end actions reflects this challenge. Sommerville and Woodward (2005) reported that, at 10 months, infants’ skill at solving cloth-pulling problems correlated with their behavior in the above-described habituation paradigm: higher skill levels were associated with greater attention to the relation between the actor and the distal goal of the observed action, whereas lower levels of skill were associated with greater attention to the relation between the actor and the means. To gain clearer evidence as to the causal relations at play, Sommerville et al. (2008) conducted an intervention study in which 10-months-old infants were trained to use a cane as a means to obtain an out of reach toy. They were then tested in a habituation paradigm analogous to the one depicted in **Figure 1**. After being trained to use the cane, infants responded systematically to the means-end goal structure in the habituation events, looking longer on new-goal trials than on new-cane trials. In contrast, infants in control conditions who received no training or only observational exposure to cane events responded unsystematically on new-goal and new-cloth trials. Moreover, the effect in the active training condition was strongest for infants who had benefitted the most from training in their own actions. That is, infants who were better at performing the cane-pulling action at the end of training looked longer to new-goal (rather than new-cane) events in the habituation paradigm test-trials. These findings indicate that success on a means-end task engenders greater sensitivity to distal goals in others’ actions. However, infants who were less successful in their own means-end actions responded randomly in the habituation task, rather than showing heightened attention to the means. Thus, it is not clear from these findings how infants perceive others’ means-end actions during the initial stages of means-end learning.

A closer look at how infants develop the ability to produce means-end actions could shed light on this early stage of learning. Infants begin to engage in well-organized means-end actions by the end of the first year. For example, Willatts (1999), following on Piaget (1954) classic studies, reported that 8-months-old infants who were presented with cloth-pulling problems like the ones in **Figure 1** would *sometimes* produce clearly intentional solutions to the problem, visually fixating the toy while systematically drawing it within reach with the cloth (see also Bates et al., 1980; Chen et al., 1997; Munakata et al., 2002; Gerson and Woodward, 2012). Early in the acquisition of a means-end action, such as tool use, infants initially focus attention on the tool or means, rather than the distal goal (Willatts, 1999; Lockman, 2000; Keen, 2011). Learning to engage in efficient means-end actions requires exploratory behavior on the tool and the object retrieved by the tool and manipulating the relations between these two. Willatts (1999) described this as a transitional progression in means-end learning: infants first focus on the means (i.e., tool) while they are learning to perform the action and only later shift their focus to the object being acted upon by the tool. Given these patterns in infants’ motor development, we might expect parallel effects on action perception. That is, when infants are at the early stages of means-end learning, their own attention to the means may lead them to focus on the relation between the actor and the means when viewing others’ means-end actions. As they begin to produce well-organized means-end actions, infants may shift their attention from the relation between the actor and the tool to the relation between the actor and the distal goal both for others’ actions and their own actions. In the current research, we examine whether this shift in focus from proximal elements of means-end problems to the distal goals of these actions seen in motor learning is paralleled in infants’ developing understanding of others’ means-end actions.

In two experiments, we investigate the specific effects of different levels of motor and observational experience on infants’ analysis of others’ means-end goals. In Experiment 1, we measure infants’ success in motor training and relate this to their action perception in a habituation paradigm in order to (1) further test the hypothesis that learning to engage in well-structured means-end actions leads to heightened attention to the relation between an actor and her distal goal, and (2) evaluate whether less successful training leads to heightened attention to the relation between an actor and the means on which she acts (i.e., the tool she first contacts). To this end, we implemented the approach developed by Sommerville et al. (2008) in their training condition, but we used a simpler means-end task (cloth-pulling) and tested younger infants (8-months-olds) in the hopes of finding greater variation in infants’ success following the training. In Experiment 2, we evaluated infants’ response to habituation events without training or with observational training as a point of comparison for the effects seen in Experiment 1.

## Experiment One

### Participants

Forty-eight 8-months-old infants (*M* age = 7.87 months; age range: 7.5–8.4 months) were included in this experiment. Infants had been born at full term (at least 37 weeks gestation) and resided in the Washington, DC, metropolitan area. All parents signed a written informed consent sheet that was approved by the University’s Internal Review Board for this research and were told their participation in the research was voluntary. Parents identified their infants’ racial group membership as follows: 46% Caucasian, 23% African American, 15% Hispanic, 8% multiracial, 2% Asian, and 6% unreported. Thirty additional infants began the procedure but were not included in the final sample due to experimenter error (*n* = 10), failure to complete the procedure due to distress (*n* = 11), failure to engage in activity during training (*n* = 3), parental interference (*n* = 3), technical errors (*n* = 2), or because they had total looking times more than 3 SDs above the sample mean (*n* = 1). The attrition rate in this study is on par with similar paradigms used with infants (e.g., Király et al., 2003; Hofer et al., 2005; Biro and Leslie, 2006; Southgate et al., 2009).

### Procedure

**FIGURE 2:**
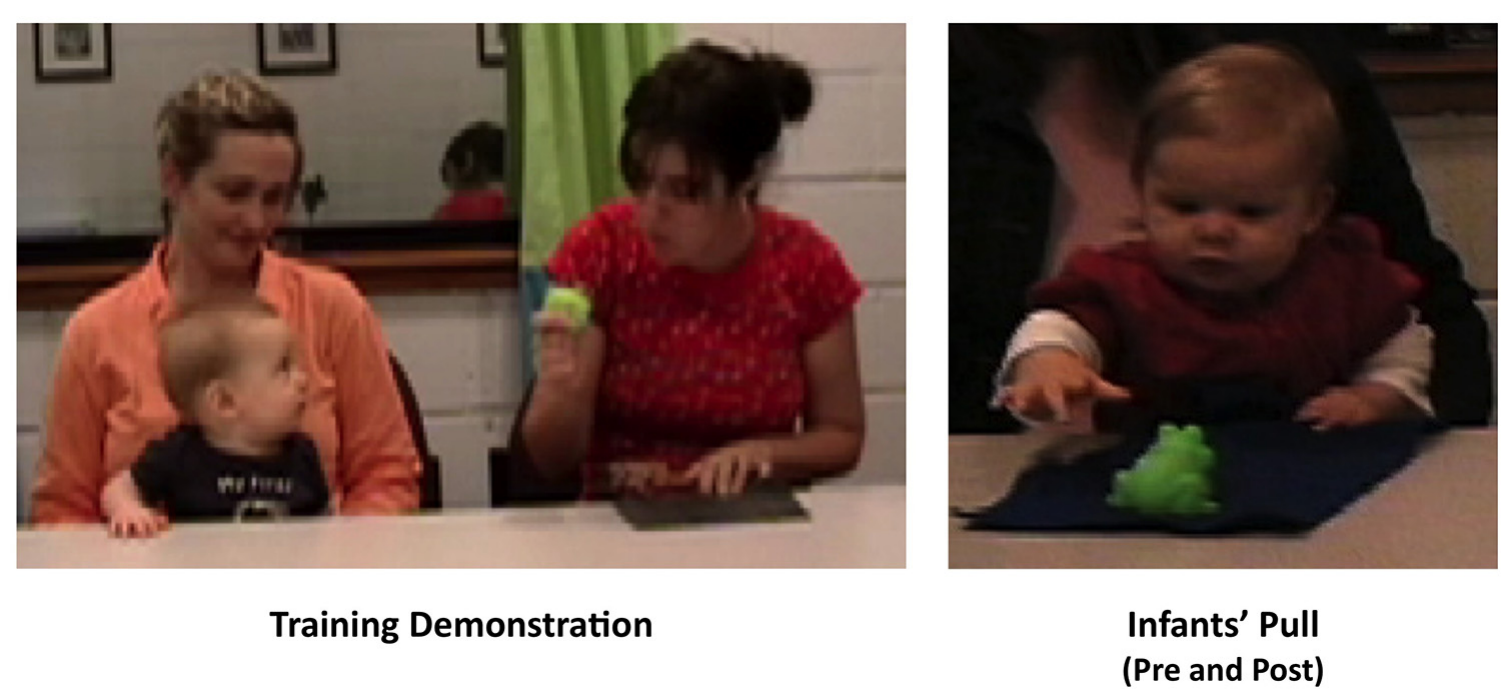
**Training session demonstration and action**.

During training, infants sat, on a parent’s lap, at a table adjusted to a height that allowed them to readily reach for and manipulate objects on its top (see **Figure 2**). Parents were asked to hold the infant securely but not to talk to the infant or influence the infants’ actions in any way. An experimenter sat next to the infant and a camera facing the infant recorded the session for later coding. Following training, infants underwent a visual habituation paradigm designed to assess their understanding of another person’s means-end action goals.

#### Active Training

The left panel in **Figure 2** depicts the events in the active training portion of the experiment. In this portion, infants were given the opportunity to act on a series of problems in which a toy was placed out of reach on the far side of a cloth that extended to within the infant’s reach. First, the infant received four *pre-training trials*. On these trials, the infant was given the chance to act on the cloth-pulling problems but was given no guidance for doing so. The experimenter set up the problem in front of the infant and then looked down at the table. She drew the infant’s attention to the cloth if necessary but did not provide more specific cues to prompt the infants’ actions. The trial ended when the infant had obtained the toy or when 30 s had elapsed. Across successive pre-training trials, infants were presented with two cloths and two toys that matched the ones they would later see in the habituation paradigm. Each cloth was presented with each of the two toys on separate trials so that each infant was presented with all possible cloth-toy combinations. The order of presentation of each toy-cloth pairing was randomized. After pre-training, the infant received five *training trials*. On these trials, the experimenter set the cloth and toy in front of herself and then enacted a means-end solution: She pulled the cloth, watching the toy as it drew near, and then retrieved the toy, inspecting it and expressing interest by saying “Ooo” as she did so. The experimenter repeated these actions twice, and then set up the same problem in front of the infant, giving the infant a chance to respond without further prompting, as on pre-training trials. Each of the five training trials involved a unique cloth-toy combination that differed from the items used during pre-training. Finally, the infant received four post-training trials, which were identical to pre-training trials. Throughout training, infants received no hands-on guidance from the experimenter or parent. All successfully completed sequences were performed by the infant him or herself.

#### Coding of Infants’ Actions

The training session was coded for the extent to which infants engaged in well-structured solutions. Infants’ actions were scored as planful if the infant maintained visual contact with the toy while pulling the cloth in one continuous movement and then retrieved or touched the toy within 3 s of the completion of the pull (see Willatts, 1999; Sommerville and Woodward, 2005). Two independent coders, each unaware of the infants’ responses in the visual habituation portion of the experiment, coded each infant’s actions. The two coders agreed on infants’ planfulness on 88% of trials (cohen’s κ = 0.76). Additional frame-by-frame coding of attention to the experimenter’s actions during training trials was assessed using a digital video coding program (Mangold, 1998). Coders measured the length of time infants attended to each aspect of the event (cloth, toy, or experimenter) during each portion of the pulling action (prior to touching the cloth, during the pull of the cloth, and during the grasp of the toy; reliability on duration of looking between two coders: *r*s > 0.95).

#### Habituation and Test

After the training procedure, infants were brought to a second testing room, equipped for the visual habituation procedure. Infants sat on a parent’s lap facing a small stage 72 cm away. On the stage sat two cloths, side by side, on a table-top surface that sloped slightly down toward the infant (so as to be easily visible but not to cause objects to slide down the slope; see **Figure 1**). Each cloth supported a different toy (a frog or a duck). A presenting experimenter (henceforth, the presenter) sat behind the stage, facing the infant. A screen was raised to hide the stage from view between trials. Parents were instructed not to talk and to look down at the infant rather than at the experimental events. A camera mounted below the stage filmed infants as they watched the events. An observer in another room watched the infant on a video monitor and coded the infant’s attention using a program that calculated looking times and habituation criteria (Casstevens, 2007). The observer could not see the experimental events and was not informed of the condition to which the infant had been assigned or the order of test trials.

At the start of each trial, the screen was lowered to reveal the stage and the presenter drew the infant’s attention by saying “Hi” and making eye contact. During habituation trials, the presenter proceeded to look down toward one of the toys, pulled the cloth toward herself and then reached toward and grasped the toy that had been drawn near. She remained still in this position, looking at the toy, until the trial ended. Infants’ attention to the event was calculated beginning as soon as the presenter had stopped moving and the trial continued until the infant had looked away for 2 consecutive seconds. When the trial ended, the screen was raised, the cloth was returned to its original position, and then the screen was lowered for the presentation of the next habituation trial. Across habituation trials, the actor consistently reached for the same cloth and toy on the same side of the table. Habituation trials were continued until the infant’s attention, summed over three consecutive trials, had declined to 50% of its initial level or for 14 trials.

Following habituation, the screen was raised and the positions of the toys on the cloths were reversed. Then the screen was lowered to allow infants to view the toys in their new positions for an infant-controlled familiarization trial. During this familiarization trial, the presenter looked down and did not look toward the stimuli. After this, the test trials were presented. On test trials, after saying “Hi” the presenter turned to grasp the near edge of one of the two cloths and look toward the toy at the end of the cloth. She then held still in this position for the duration of the trial, which was infant-controlled, as during habituation. It is important to note that, unlike in the habituation trials, during test trials the presenter never moved the cloth or touched the toy (matching the procedure used in Sommerville and Woodward, 2005). On *new-goal trials*, she grasped the same cloth that she had acted on during habituation, which now supported a new toy at its far end. On *new-cloth trials* she grasped the cloth she had not acted on during habituation, which held her prior goal toy at its far end. Three new-goal and new-cloth trials were presented in alternation. The type of test trial seen first, the side to which the presenter reached during habituation, and the toy that was the presenter’s goal during habituation were counterbalanced across infants.

Each infant’s video session was coded after the fact by a second independent observer. The online and reliability observers were counted as agreeing if they agreed on the point at which the infant looked away to end the trial. The two observers agreed on the endpoints of 95% of test trials. To evaluate potential observer bias, all disagreements were categorized as those that would indicate bias in favor of the hypothesis on the part of the on-line coder versus those that would indicate bias against the hypothesis. The observers’ disagreements were randomly distributed (Fisher’s Exact Test, *ns*).

### Results

#### Training Session: Assessment of Quality of Motor Training

Coding of infants’ attention to the experimenter’s actions during training trials indicated that infants attended to the relevant aspect of the action during the majority of the experimenter’s actions throughout training trials. That is, they attended to the cloth during the pulling action (90% of the time on average) and to the toy and experimenter during the grasping action (83% of the time on average).

**FIGURE 3:**
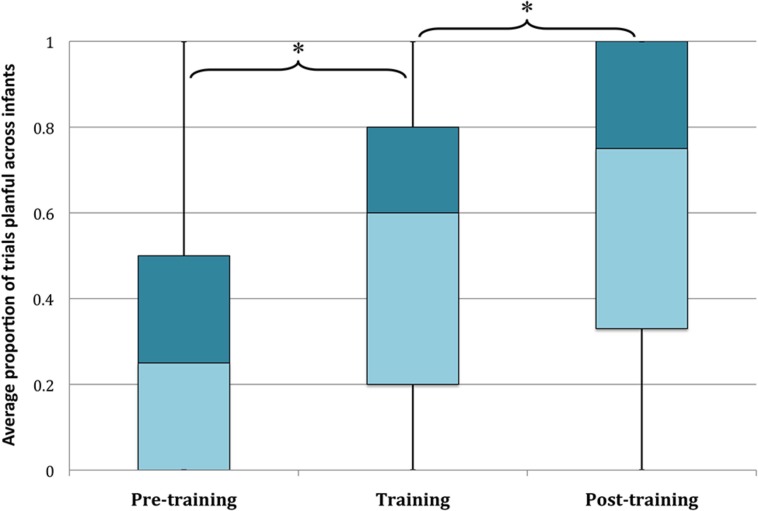
**Infants in the active condition increased in planfulness from pre-training to post-training (box plot median, quartiles, minimum, and maximum proportion of trials within each portion of training that infants were planful) ^∗^*p* < 0.02**.

On the 13 training trials (including pre- and post-training trials), infants produced planful actions on an average of 6.40 (SEM *=*0.51) trials overall. As shown in **Figure 3**, infants increased their planfulness from pre- to post-training trials. A repeated measures analysis of variance (ANOVA) on the proportion of planful actions in the pre-training, training, and post-training trials revealed a significant increase in planful actions across these phases, *F*(2,45) = 18.13, *p*< 0.001, $\eta_{p}^{2}$ = 0.45. Pairwise comparisons of the estimated marginal means indicated that infants’ planfulness increased significantly from pre-training to training (mean difference = 0.23, SEM = 0.048; *p*< 0.001) and from training to post-training (mean difference = 0.14, SEM = .05; *p* = 0.013). Age did not correlate reliably with infants’ degree of planfulness in any of the three phases or with infants’ degree of improvement from pre-training to post-training (all *r*s < 0.12, *p*s > 0.42). Thus, the active training procedure reliably increased the extent to which infants engaged in well-organized means-end actions. Even so, infants’ responses to training varied. Only half of the infants were highly planful after training—25 of the 48 infants achieved planful scores on 3 or 4 out of the 4 post-training trials. Thus, a median split of planfulness (Quality of Training) corresponded with a theoretically meaningful cutoff.

Planful and unplanful infants did not differ from one another in age (*p* = 0.89). We further assessed whether planful and unplanful infants differed in their general attention to assure that unplanful infants were not simply less alert in general. Infants in the two groups did not differ in amount of attention (i.e., the length of looking to each trial) at the beginning (*p* = 0.33) or end of habituation trials (*p* = 0.98). Further, the number of habituation trials needed to reach habituation criterion (often thought of as a measure of speed of processing and known to be related to later intelligence; Fagan, 1992) did not differ between the two groups (*p* = 0.38). Planful and unplanful infants did not differ in overall amount of attention during test trials (collapsed across two different kinds of test-trials) during test-trials (*p* = 0.39). Finally, we also assessed infants’ attention during the training session in order to assure that infants had the same opportunity to learn from training trials. Infants in the two groups spent comparable amounts of time attending to the relevant aspects of the experimenter’s actions during training trials during both the pulling (*p* = 0.24) and grasping (*p* = 0.33) portions of training trials. Across groups, no significant correlation was found between attention to relevant aspects of training and post-training planfulness (*r*s < 0.23, *p*s > 0.13). Thus, there was no evidence that variations in infants’ attentiveness during the procedure, or in their age, accounted for their ability to benefit from training (see **Table 1** for a summary of means and SDs). Subsequent analyses took the variation in the extent to which infants benefited from training into account, as described below.

**Table 1:** Similarity in infants’ attentional patterns across active groups.

	Age	Attn to beg of Hab	Attn to end of Hab	# of Hab trials	Total attn to test trials	Pre-training planfulness	Attn to pull (in training)	Attn to grasp (in training)
Below median planful *M*(SEM)	7.86 mos (0.06)	46.69 s (6.83)	14.04 s (1.87)	8.35 (0.58)	40.66 s (4.91)	0.23 (0.06)	2.48 s (0.09)	2.33 s (0.15)
Above median planful *M*(*SEM*)	7.85 mos (0.05)	39.08 s (3.54)	13.97 s (1.39)	9.08 (0.59)	35.39 s (3.74)	0.33 (0.06)	2.63 s (0.07)	2.53 s (0.14)
*t*-test *p*-value	0.89	0.33	0.98	0.38	0.39	0.24	0.33	0.36

#### Habituation Session: Relative Attention to Cloth and Goal Relations

Preliminary analyses assessed infants’ attention during the habituation trials. A repeated measures ANOVA with habituation trial (the first three and last three trials for each infant) as the repeated measure revealed a main effect of trial, *F*(1,47) = 65.11, *p*< 0.001, $\eta_{p}^{2}$ = 0.58, reflecting a decline in attention across trials. Infants required ∼9 trials on average to reach habituation criteria.^1^

**FIGURE 4:**
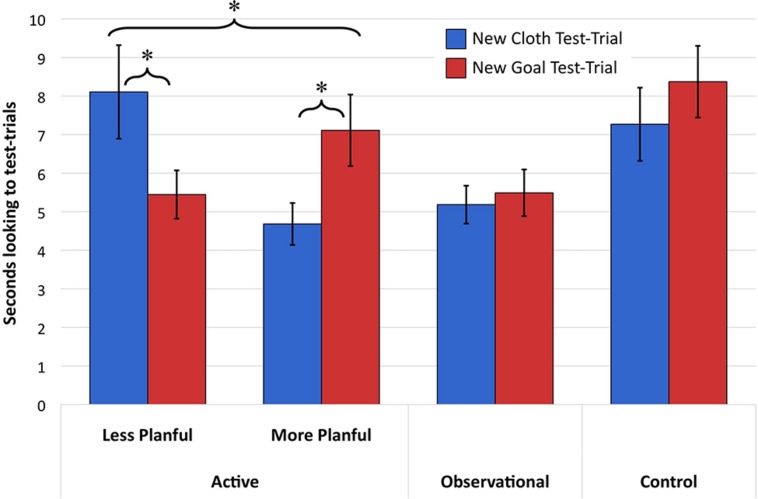
**Mean looking times SEs to test-trial events across conditions ^∗^*p* < 0.02**.

The focal analysis concerned infants’ differential attention to the change in relation between the agent and the means she used (i.e., new-cloth test events) or her distal goal (i.e., new-goal test events) and whether differential responses to these test events varied as a function of the success of training. A repeated-measures ANOVA was conducted with average looking time to the new-goal and new-cloth events as the repeated measure (Type). In order to take into account the variability in training success, a median split of infants’ planfulness at the end of training (Training Success) was included as a between-subjects factor. As discussed above, approximately half of the infants were successful in planfully carrying out the means-end action in at least three of the four post-training trials and these two groups did not differ in age, attention during habituation, or attention during training trials. This analysis revealed a significant Type X Training Success interaction, *F*(1,46) = 14.50, *p*< 0.001, $\eta_{p}^{2}$ = 0.24. The main effects of Type and Training Success were not significant (*F*s < 0.75)^2^. Pairwise comparisons of the estimated marginal means (see **Figure 4** for raw means and standard errors) revealed that infants below the median in planfulness at post-training looked significantly longer to new-cloth than to new-goal trials (mean difference = 2.66, SEM = 0.96; *p* = 0.008) whereas infants above the median in planfulness looked significantly longer to new-goal than new-cloth trials (mean difference = 2.43, SEM = 0.92; *p* = 0.012).

#### Relations Between Training Success and Action Perception

Given the differences found based on the success of training as reflected in the median split of post-training activity (training success), we further explored the relation between planfulness in different phases of training and looking time differences in the habituation paradigm. Infants’ planfulness in post-training was unrelated to their planfulness in pre-training (*r* = 0.15, *p* = 0.32), suggesting that individual differences in post-training planfulness were not a function of motor abilities prior to training. We also examined whether attention to different aspects of the experimenter’s actions during training trials related to infants’ new-goal preference in the habituation phase, but no aspect of attention was significantly related (*r*s < 0.24, *p*s > 0.11).

To examine the unique contribution of pre versus post-training on infants’ differential looking to new- versus old-goal test events, we performed a hierarchical multiple regression analysis. For each infant, we calculated a difference score reflecting his or her relative visual preference for new-goal trials compared to new-cloth trials (average looking time on new-goal trials minus average looking time on new-cloth trials; see Sommerville et al., 2005 for a similar measure) and entered this as the dependent variable. Pre- and post-training planfulness were entered in two steps. In step 1, pre-training planfulness was the independent variable. In step 2, post-training planfulness was added to the step 1 equation.

The results of step 1 indicated that the variance accounted for by pre-training planfulness was not significantly different from zero (see **Table 2**). Adding post-training planfulness as a predictor significantly improved the model; post-training planfulness was positively related to new-goal preference (*B* = 0.15), whereas pre-training planfulness was unrelated (*B* = -0.01). Thus, infants’ learning from the training session (as evidenced in post-training planfulness), rather than their starting means-end abilities, predicted their subsequent responses to the observed actions in the habituation paradigm.

**Table 2:** Hierarchical multiple regression: effect of post-training planfulness on new-goal preference.

Variables	Model 1	Model 2
Intercept	0.51^∗∗^	0.42^∗∗^
Pre-training planfulness	0.02	–0.01
Post-training planfulness		0.15^∗^
Adjusted *R*^2^	–0.022	0.054
Model *F*	0.021	2.31
Change *R*^2^	<0.001	0.95^∗^
Incremental F		4.6^∗^

^∗^*p* = 0.037, ^∗∗^*p* < 0.001.

### Discussion

The findings of Experiment 1 converge with those of Sommerville et al. (2008) in showing that training in a means-end action supports infants’ sensitivity to others’ means-end actions. Infants who benefited from means-end training, in that they became able to organize their own actions in service of a distal goal, subsequently responded to the higher-order goal structure of another person’s means-end actions. Extending beyond the findings of Sommerville et al. (2008), the current findings also indicate that infants who were less successful in organizing their means-end actions attended, instead, to the relation between the observed agent and the means she acted on initially (the cloth). Importantly, there was no evidence that infants who did less well in organizing their own actions were less alert, attentive, or engaged than infants who were more successful. Further, both planful and unplanful infants responded systematically on test trials, showing that they had encoded and remembered the habituation events. The differences in infants’ responses to the test events were not predicted by infants’ ability to perform the task prior to training, but rather were predicted by infants’ skill following training. Thus, the differential findings based on planfulness seemed to reflect infants’ experiences during the training phase, rather than differential abilities they brought with them into the laboratory.

The coding scheme by which infants’ actions were identified as planful gives some clues as to the reason infants who did not benefit showed a bias toward interpreting the means-end action as directed at the simple one-action-step goal (i.e., the cloth). Action task trials in which the infant looked at the cloth during a pull rather than the toy or in which the child pulled the cloth but then failed to retrieve the toy within a short amount of time were qualified as unplanful (and the child often did not attain the toy at the end of the trial). Those infants who produced more of these actions during training may have spent more time attending to the cloth than other infants in that they concentrated their attention on the cloth in service of attempting to successfully coordinate their actions on the cloth. These patterns are consistent with developmental patterns in infant motor development: When initially learning new actions, infants seem first to attend to the means of the action and as they gain proficiency, they shift attention to the goal (Willatts, 1999).

Our findings raise the question of why infants varied in their learning from the training manipulation. This variation was not accounted for by age. It is likely instead that infants’ ability to benefit from motor training depended on their existing motor abilities and developmental readiness for learning (see Piaget, 1954; Lockman, 2000; Keen, 2011). The current findings do not provide evidence for fully evaluating this issue. Further research is needed to investigate the developmental predictors of motor learning and their relation to generalizing motor information to the perception of others’ actions.

What is it that self-produced experience provided for infants in this experiment? One interesting possibility is they learned about the goal of the action through simply observing their own actions. We know that infants this age can learn about the goals of tool-use actions without active experience organizing their own actions on tools in certain circumstances (see, for example, Gerson and Woodward, 2012, 2014c). On the other hand, there is reason to think that the act of producing an action, rather than simply observing it, may be particularly informative because it yields shared action perception representations.

Recent research has investigated the unique effects of active, relative to observational, experience on action perception (Sommerville et al., 2008; Gerson and Woodward, 2014a; Gerson et al., 2014), face perception (Libertus and Needham, 2011, 2014), and spatial perception (Frick and Wang, 2014). If the mirror system plays a role in the link between motor experience and action perception (see Hunnius and Bekkering, 2014; Woodward and Gerson, 2014, for discussion), benefits should (at least initially) be unique to active experience as this widens the motor repertoire of the infant.

Accordingly, recent studies have examined the effects of observational experience with novel actions in training studies with infants matching those described above. Gerson and Woodward (2014a) found that active, but not observational, training with reaching actions led 3-months-old infants to recognize the goal of a grasping action in a habituation paradigm. Similarly, Sommerville et al. (2008) included an observational training condition in their means-end training experiment, in which 10-months-old infants watched an experimenter produce planful actions using the same canes and for the same number of trials as infants in the active training condition. Infants in this condition did not respond systematically in the habituation paradigm. Thus, these findings indicate that self-produced actions provide stronger support for viewing others’ actions as goal-directed than do observed actions. In the Sommerville et al. (2008) experiment, all infants in the observational condition received a set amount of experience observing means-end actions (the mean of that produced by actively trained infants), so effects of variability in the amount of observational experience received could not be assessed (but see Sommerville et al., 2011).

In Experiment 2, we matched the amount of experience watching means-end actions to that of a yoked infant in the active condition from Experiment 1. Given the differential effects of training found in Experiment 1, we aimed to examine whether different amounts of observational experience would differentially influence action perception. That is, would the amount of observational experience received influence infants’ relative attention to the relation between the actor and the means or goal of her actions, as it did in Experiment 1? This matched variability allows us to assess potential correlations between observed activity and action perception. We also included a control condition in which infants had the chance to explore each cloth and toy prior to the habituation paradigm but never got to act on one in relation to the other and did not observe this action prior to the habituation paradigm. This allowed us to compare observational experience with infants’ action perception when they received no means-end training.

## Experiment Two

### Participants

Forty-eight 8-months-old infants (*M* age = 7.9 months; age range: 7.27–8.43 months) participated in one of two conditions in this experiment: observational or control. Infants had been born at full term (at least 37 weeks gestation) and resided in the Washington,DC, metropolitan area. Parents identified their infants’ racial group membership as follows: 60% Caucasian, 3% Asian, 17% African American, 10% Hispanic, and 10% multiracial. Twenty-nine additional infants began the procedure but were not included in the final sample due to experimenter error (*n* = 13), failure to complete the procedure due to distress (*n* = 12), parental interference (*n* = 1), or because they had total looking times more than 3 SDs above the sample mean (*n* = 3).

### Procedure

Infants underwent a “training” period prior to participation in the habituation paradigm. During this session, as in Experiment One, infants sat on a parent’s lap at a table adjusted to a height that allowed them to readily reach for and manipulate objects on its top. Parents were asked to hold the infant securely but not to talk to the infant or influence the infants’ actions in any way. An experimenter sat next to the infant and a camera facing the infant recorded the session for later coding.

#### Observational Training

Infants in the observational condition were shown the same series of cloth-pulling problems as infants in the active condition from Experiment One, but they observed the experimenter solving each problem and were not given the opportunity to act on the toys themselves. To equate, as much as possible, the duration of training in this condition to the amount of experience received in the active condition from Experiment One, the duration of each trial for infants in the active condition was coded, and the session from each infant in the active condition was used to generate a script for an infant in the observational condition that specified the duration of each observation trial. Because the experimenter’s actions were generally more well-organized than those of the infants, this meant that the experimenter sometimes repeated the problem several times in order to keep the infant engaged for the full trial duration. Thus, infants in the observational condition had equivalent durations of exposure to the problems as did infants in the active condition from Experiment One, and they viewed more instances of well-organized solutions than did infants in the active condition (see below for details).

#### Control “Training”

Infants in the control condition were given the opportunity to explore each cloth and each toy that were involved in the active and observational training, but they saw each cloth and each toy presented independently (i.e., sequentially), rather than in the context of a means-end problem. The order of presentation paralleled the order in the active and observational conditions, with infants first being given each of the four items involved in the pre- and post-training phase for 15 s each, then each of the 10 items from the training phase for 30 s each, and then the four pre- and post-training items again for 15 s each.

#### Coding of Training Session

Videos of the observational condition were coded for infants’ attention during each phase of the experimenter’s movements–grasping the cloth, pulling the cloth, and retrieving the toy—to identify the number of complete means-end actions that each infant viewed. To assess reliability, a second independent coder coded the sessions for 25% of infants. The two coder’s judgments of the number of planful actions infants observed in each phase of the training session were highly correlated, *r* = 0.99. As in the training trials from Experiment One, additional frame-by-frame coding of attention to the experimenter’s actions during observational training was assessed using a digital video coding program (Mangold, 1998; reliability: *r*s > 0.95).

#### Habituation and Test

The habituation procedure in this experiment was identical to that of Experiment One. Reliability of the online coders was assessed and coders agreed on the end of the trials for 94% of test trials (cohen’s κ = 0.88). To evaluate potential observer bias, all disagreements were categorized as those that would indicate bias in favor of the hypothesis on the part of the on-line coder versus those that would indicate bias against the hypothesis. The observers’ disagreements were randomly distributed (Fisher’s Exact Test, *ns*).

### Results

#### Training Session: Assessment of Amount of Observational Training

In the observational condition, we examined the number of planful pulls observed by each infant. Because the experimenter repeated planful pulls for the duration of each trial in the observational condition, infants in this condition had the opportunity to view more planful actions than infants in Experiment One produced (mean number of planful pulls in Experiment One was 6.4, ranging from 0 to 12). Coding of infants’ attention to the pulls revealed that infants in the observational condition viewed 24 planful pulls on average (range = 16–29). Further, frame-by-frame coding of infants’ attention to the experimenter’s actions indicated that they attended to the relevant aspect of the action the majority of the time: to the cloth during pulling actions (88% of the time) and to the toy and experimenter during the grasping action (77% of the time). Infants in the observational condition did not differ from infants in the active condition from Experiment One in their attention to any of these aspects (*p*s > 0.10).

#### Habituation Session: Relative Attention to Cloth and Goal Relations

Preliminary analyses assessed infants’ attention during the habituation trials. A repeated-measures ANOVA with the first three and last three trials of habituation as repeated measures and condition (observational versus control) as a between subjects factor revealed a main effect indicating a significant decrease in attention across conditions, *F*(1,46) = 97.04, *p*< 0.001, $\eta_{p}^{2}$ = 0.68, no interaction between condition and trials (*p*> 0.57), and no main effect of condition (*p* > 0.49). When the active condition from Experiment One was included in this analysis there was again no interaction between condition and trial. Infants in Experiment Two habituated in approximately eight trials on average.

The main analysis concerned whether infants in either the observational or control condition showed preferential looking to the new-goal or new-cloth test-trials and whether they differed from each other and/or infants in the active condition from Experiment 1 who were more or less planful at the end of training. We first examined only the infants in the control and observational conditions (see **Figure 4**). A repeated-measures ANOVA with test-trial type as the repeated measure (new-goal or new-cloth) and condition as the between subjects factor (observational or control) revealed no main effect of Type [*F*(1,46) = 1.58, *p* = 0.22, $\eta_{p}^{2}$ = 0.03] and no interaction between Condition and Type [*F*(1,46) = 0.51, *p* = 0.48, $\eta_{p}^{2}$ = 0.01]. A main effect of condition [*F*(1,46) = 7.10, *p* = 0.01, $\eta_{p}^{2}$ = 0.13) indicated that infants in the control condition looked longer across test trials than did infants in the observational condition.

#### Relations Between Amount of Training and Action Perception

As a measure of experience in the observational condition, we also examined whether the number of planful pulls observed differentially influenced looking times to different test trials. As a measure of the possible continuous relation between experience observing planful pulls and new-goal preference (as found in Experiment 1), we calculated the difference between average new-goal and new-cloth (*average difference*) trials and examined its relation to number of pulls observed. No significant relation was found (*r* = 0.085, *p* = 0.69). As in Experiment One, we also examined whether any aspect of attention to the experimenter’s actions during training trials related to new-goal preference in the habituation paradigm. No significant relations were found (*r*s < 0.28, *p*s > 0.20).

Because the number of pulls presented (and thus the maximum possible number to observe) was randomly assigned to infants based on scripts from activity of infants in Experiment One, we also took into account individual differences created by the infants themselves by dividing the number of trials observed by the number of trials presented. On average, infants observed 89% of the actions produced by the experimenter (range: 63–100%). The relation between proportion of pulls observed and new-goal preference was not significant (*r* = -0.25, *p* = 0.24).

As in Experiment 1, we created a median split of experience (Amount of Training: more or fewer than 15 planful pulls observed). In order to compare the effects of experience in the observational condition with infants from the active condition in Experiment One directly, we conducted a repeated-measures ANOVA with test-trial type as the repeated measure and condition (active or observational) and Amount of Training as between subjects factors. This revealed no main effects of Type (*F*< 0.05, *p* > 0.85), Condition (*F* < 1.4, *p*> 0.24), or Amount of Training (*F* < 1.3, *p*> 0.27), and no interaction between Type and Condition [*F*(1,68) = 0.16, *p* = 0.69, $\eta_{p}^{2}$ = 0.002]. A significant interaction between Type and Amount of Training [*F*(1,68) = 5.74, *p* = 0.019, $\eta_{p}^{2}$ = 0.078] was qualified by a three-way interaction between Type, Condition, and Amount of Training [*F*(1,68) = 5.67, *p* = 0.02, $\eta_{p}^{2}$ = 0.077]. Comparisons of estimated marginal means again revealed that the three-way interaction was a function of the significant effect of Type that was in opposite directions for infants above and below the median in active experience in Experiment One (as described above; *p*s ≤ 0.005) but no significant differences between Type in either infants above (estimated marginal means for new-cloth trials, 5.69. SEM = 1.24, and new-goal trials, 6.00. SEM = 1.16) or below the median in experience (estimated marginal means for new-cloth trials, 4.82. SEM = 1.05, and new-goal trials, 5.12. SEM = 0.98) in the observational condition (mean differences < 0.32, *p*s > 0.79).

Given the lack of effects of experience in the observational condition and the lack of difference between observational and control conditions, we then collapsed across these conditions to examine whether responses in the habituation paradigm differed between these conditions and the more and less planful infants from the active condition in Experiment One. We conducted a univariate ANOVA with proportion of attention to the new-goal test trials (new-goal)/(new-goal + new-cloth) as the dependent variable and condition group (active-high, active-low, or observational and control) as the between-subjects factor. The condition groups significantly differed from one another in new-goal preference [*F*(1,2) = 5.72, *p* = 0.005, $\eta_{p}^{2}$ = 0.11], and we followed up with planned comparisons between conditions. Pairwise comparisons of the estimated marginal means indicated that infants in the observational and control conditions had significantly higher new-goal preferences than unplanful infants in the active condition (mean difference = 0.08, SEM = 0.04, *p* = 0.03) and had marginally lower new-goal preferences than planful infants in the active condition (mean difference = 0.06, SEM = 0.04, *p* = 0.095; see **Figure 4**).

### Discussion

The results of Experiment 2 did not reveal effects of observational experience with means-end on infants’ action perception. This result is consistent with findings indicating that observation of means-end training is not as beneficial as active training at 10 months of age (Sommerville et al., 2008). In this experiment, we expanded on prior research to explore individual differences in the amount of observational experience received. Given the importance of the amount of planful actions produced in the active condition in Experiment 1, we allowed infants the opportunity to observe planful actions for the same range of time as infants in the active condition. This way of matching infants meant that infants in the observational condition experienced more instances of well-formed, planful actions than did infants in the active condition. Even so, infants in the observational condition did not demonstrate a benefit from training at a group level or show any of the same patterns of individual differences as infants in Experiment 1.

It is important to note that the difference in effects between conditions cannot be due to a difference in opportunities to observe or infant attentiveness. In creating yoked observational scripts, we erred on the side of allowing infants in the observational condition to view more demonstrations than their partners in the active condition. Infants in the observational condition always saw more planful actions during the training phase than their matched partner in the active condition from Experiment 1 (in fact, infants in this condition saw almost four times more exemplars of the cloth-pulling action than their active training counterparts). They attended to cloth-pulling actions for as long as infants in the active condition and thus received equal exposure to the toys and cloths. The physical causal information (pulling the cloth makes the toy move) was identical to infants across the active and observational conditions. Looking times during both the habituation and test phases of the looking time procedure did not indicate any differences in overall attention to the events between the two studies. Additionally, the patterns found in the active condition were a result of attention to a specific relation between particular actions, means, and objects, rather than general attention to the event or a particular toy or cloth.

Despite controlling for attention, there may be other differences in readiness to learn that we could not assess in our observational condition. The yoking procedure we used randomly assigned infants in the observational condition to a script duration based on an active infants’ timing. It is possible that seeing a greater number of demonstrations at a faster rate than infants in the active condition could have hindered some infants’ ability to make sense of the viewed action, but we had no way to take into consideration the length of time that each individual infant in the observational condition may have needed to benefit from observation. Thus, it is possible that infants tested under conditions in which readiness to learn is taken into consideration may show greater benefits from observation.

Critically, however, the design of the current experiment parallels a real-world difference between active and observational learning. In active learning, infants’ experience is self-generated and thus can be readily calibrated to their current learning state (e.g., infants can continue to act on the world until they have all the information that they need to learn relevant information). In contrast, when learning via observation, infants are at the behest of the caregiver, adult or more advanced peer who is doing the demonstrating. During observational learning, it is the demonstrator that decides how much information to give infants and for how long; given that demonstrators do not have direct access to infants’ knowledge base or learning state (although infants may provide implicit cues to this state), information accrued via observation may be less well suited to an infant’s learning state than is information accrued via active learning. Indeed, this distinction between active and observational learning may be one of the factors driving the potential benefits of active versus observational learning. Future work can directly assess this possibility.

The observational and control conditions provided a point of comparison for the active training groups, allowing us to examine whether both the low and high planful groups differed from how infants might respond to the habituation events spontaneously. The fact that both groups of active infants differed from the observational and control conditions in opposite directions suggests that initial, unsuccessful attempts at means-end problems push attention to the proximal agent-means relation whereas more successful training pushes attention to the distal agent-goal relation.

## General Discussion

Prior findings have shown that active motor experience affects infants’ sensitivity to the goal structure of others’ simple actions (Sommerville et al., 2005; Gerson and Woodward, 2014a,b). Our question, in the current studies, was whether active motor experience also supports infants’ emerging sensitivity to others’ distal goals. Understanding distal goals requires that the perceiver look beyond the actor’s immediate motor interactions in order to consider his or her potential distal goals, and this raises the question of whether and how concrete motor experiences could contribute to this aspect of goal analysis. Our findings provide evidence that active motor experience supports infants’ analysis of distal goals, and further, provide new insight into the influence of infants’ motor experiences on their analysis of others’ actions.

In the current experiments, infants saw a chain of interrelated actions in the habituation trials of the looking time paradigm. The presenter first reached for and grasped a cloth. After pulling on it, she then reached for and grasped the toy at the end of the cloth. Test trials assessed whether infants viewed the experimenter’s actions on the cloth as directed at the cloth itself, or instead as directed at the toy. The findings of Experiment 1 indicated that infants’ active experience in a cloth-pulling task predicted which of these interpretations they adopted. Infants who benefited from training and became highly organized in their own actions viewed the experimenter’s action on the cloth as directed at the toy. Infants who were less successful in their training activities viewed her actions as directed at the cloth. Compared to infants in Experiment 2, who underwent observational training or no training, infants in Experiment 1 showed systematic differences in each response pattern. Thus, at a first level of analysis, the current findings contribute support to the conclusion that infants’ interpretation of distal goals is influenced by their own motor experience (Sommerville and Woodward, 2005; Sommerville et al., 2008).

The current findings go beyond prior work in demonstrating that variation in infants’ success in means-end activities leads to systematic variation in their analysis of others’ actions. Infants who benefited from active training showed the higher-level interpretation of the events in the habituation paradigm, consistent with findings from older infants (Sommerville et al., 2008). But infants who engaged in ineffective means-end actions showed just the opposite response, interpreting the observed actions in terms of the proximal goal (the cloth) rather than the distal goal. These distinct patterns of response mirror the patterns that occur during developments in infants’ own means-end actions (Willatts, 1999). This result suggests that the processes that give rise to means-end structure in infants’ motor behavior also support the emergence of means-end structure in their analysis of others’ goals.

We can conclude, then, that there is a specific relation between organizing means-end action toward the goal and understanding others’ means-end actions as organized toward a goal. The individual differences found in Experiment 1 suggest that infants may at first concentrate and learn about the means of a multi-step action and then change their focus to the goal once they gain proficiency with a new action. Active experience seems to focus infants’ attention on relevant relations and, depending on the nature of their own actions, this could be the relation between the cloth (i.e., proximal goal) and the agent or the goal (i.e., distal goal) and the agent.

Importantly, this shift in focus was not seen in Experiment 2, when infants observed an adult engage in repeated, well-structured means-end actions, nor was there any indication that variations in observational experience related to variations in infants’ responses to the habituation events. Infants’ failure to benefit from the observational training is striking. In the observational training, infants were witness to critical information about the goal-structure of the cloth-pulling action. They viewed the causal relation between acting on the cloth and attaining the toy, and they saw the experimenter express interest in the toy. Infants were highly attentive to these events, and yet seemed not to recover meaningful information from them regarding the goal structure of cloth-pulling events. This finding, in conjunction with previous research (Sommerville et al., 2008; Gerson and Woodward, 2014a), suggests that active experience provides a particularly potent, and possibly unique, source of evidence for understanding others’ actions during early development.

Even so, open questions remain concerning the nature of the benefit conferred by active experience. It is possible that self-produced actions yield information about goal structure that infants cannot glean from observation alone. Alternatively, it remains possible that infants can glean goal information from observational experience, but were unable to demonstrate it given the demands of the current task. The training and habituation sessions were conducted in different rooms and involved different people, and infants have difficulty carrying goal information across contexts (Sommerville and Crane, 2009). Thus, active experience may create particularly robust or “portable” representations, as compared to observational experience (see Gerson and Woodward, 2010 for further discussion).

The current findings indicate that infants’ own actions render changes in their sensitivity to the goal structure of others’ actions. Recent findings in infants (van Elk et al., 2008; Southgate et al., 2009; Saby et al., 2012; Gerson et al., 2014; Cannon et al., 2015) suggest that the motor system is active during, and may play a role in, infants’ perception of others’ actions. Although the current findings do not provide direct evidence concerning the neural mechanisms at work, they raise the question of whether shared neurocognitive representations support infants’ analysis of higher-order goals. Mirror neurons in primates and mirror systems in humans are modulated, not only by the goals of simple actions, but also by overarching goals that structure action sequences (Fogassi et al., 2005; Iacoboni et al., 2005). For example, Fogassi et al. (2005) found mirror neurons in macaque monkeys that fired differentially to grasping actions that preceded eating versus placing of the grasped object when there were contextual cues to support one of these two analyses of the grasp. In this way Rizzolatti and Craighero (2004) suggest that “chains” of neurons in the inferior parietal lobe could facilitate action understanding through linking sequences of actions and goals (see also Sinagaglia, 2009). Similar results have been found with human adults (Iacoboni et al., 2005). These findings suggest that there might be shared representations at higher-order levels that could play a role in linking active experience and action understanding. Thus, it is plausible that these representations may emerge in development and support early developments in action understanding. Clearly, further research is needed to investigate this possibility.

These open issues aside, the current findings support the notion that self-produced experience is uniquely beneficial for action perception in the first year of life. They shed light on the nature of information gained from active experience with means-end actions, indicating that the shift in one’s own attention to the means or distal goal when learning to produce multi-step actions is similarly reflected in infants’ perception of others’ means-end actions.

## Conflict of Interest Statement

The authors declare that the research was conducted in the absence of any commercial or financial relationships that could be construed as a potential conflict of interest.

## Footnotes

1 Four infants reached 14 habituation trials without meeting the habituation criterion. When these infants were removed from the sample, the principle findings were unchanged. Therefore the analyses are reported for the full sample.

2 We conducted this critical analysis with a randomly selected subset of 24 subjects (in order to match the sample size of infants in the observational and control conditions of Experiment 2) and saw a nearly identical pattern: Type X Training success: *F*(1,22) = 10.20, *p* = 0.004; no significant main effects (*F*s < 2.70).
